# Waiting Time for Coronal Preparation and the Influence of Different Cements on Tensile Strength of Metal Posts

**DOI:** 10.1155/2012/785427

**Published:** 2012-01-15

**Authors:** Ilione Kruschewsky Costa Sousa Oliveira, Ynara Bosco de Oliveira Lima Arsati, Roberta Tarkany Basting, Fabiana Mantovani Gomes França

**Affiliations:** Departamento de Odontologia Restauradora-Dentística, Faculdade de Odontologia e Centro de Pesquisas Odontológicas São Leopoldo Mandic, Rua José Rocha Junqueira, 13045–755 Campinas, SP, Brazil

## Abstract

This study aimed to assess the effect of post-cementation waiting time for core preparation of cemented cast posts and cores had on retention in the root canal, using two different luting materials. Sixty extracted human canines were sectioned 16 mm from the root apex. After cast nickel-chromium metal posts and cores were fabricated and luted with zinc phosphate (ZP) cement or resin cement (RC), the specimens were divided into 3 groups (*n* = 10) according to the waiting time for core preparation: no preparation (control), 15 minutes, or 1 week after the core cementation. At the appropriate time, the specimens were subjected to a tensile load test (0.5 mm/min) until failure. Two-way ANOVA (time versus cement) and the Tukey tests (*P* < 0.05) showed significantly higher (*P* < 0.05) tensile strength values for the ZP cement groups than for the RC groups. Core preparation and post-cementation waiting time for core recontouring did not influence the retention strength. ZP was the best material for intraradicular metal post cementation.

## 1. Introduction

The cast metal core is a component frequently used to restore endodontically treated teeth with extensive coronal destruction [1, 2]. Post retention in the root canal is fundamental for the longevity and success of treatment. Length, shape, diameter, and post surface, as well as the type of cement used, are factors that may affect core retention and stability. A prefabricated retainer must adapt adequately to the prepared root canal; otherwise, a cast post and core should be the treatment option [3].

There is no consensus with regard to the choice of a luting agent for cast metal cores; therefore, the choice of cement must take into consideration its biological and functional properties [4, 5]. An ideal luting agent must be sufficiently fluid to flow, provide adequate working and setting time to allow the part to seat, permit easy removal of excess material, and allow adjustments to be made to the part without losing retention. Cements can retain the core in the root canal mechanically, chemically, or by other means. Mechanical bonding is not always effective for retention, and a cement with the potential to chemically bond to tooth surfaces and prosthesis may be indicated [6].

In addition to being the material most frequently used for this purpose, zinc phosphate cement is the oldest of the luting agents; therefore, it serves as a standard for comparison with new systems [7–9]. It does not bond chemically but bonds only by mechanical interlocking at the interfaces. It has high resistance and forms a thin film; it can support elastic deformations even when it is used as a luting agent for restorations subject to high masticatory stresses [10].

More recently, resin cements have become an alternative to zinc phosphate cement, particularly because they are practically insoluble in the oral medium and have adhesive properties [1, 9, 11, 12]. Dual-activated cements provide long working time until they are exposed to light activation, and, due to the chemical polymerization process, their bond strength continues to increase over time [13]. However, after posts have been cemented with resin agents, stresses produced by polymerization shrinkage may harm the integrity of the bond between the resin cement and the root canal walls [1]. The use of sodium hypochlorite as an irrigant solution during endodontic treatment, as well as filling cements containing eugenol in their formulae, may also influence retention of posts cemented with resin cement [14–16]. Some studies have concluded that cores cemented with zinc phosphate cement have retention values higher than or similar to those cemented with resin cements [17–20]. However, other studies have shown better performance of resin cements when compared with zinc phosphate cement [21].

Amongst the various reasons for failure in teeth restored with a core, detachment, displacement, and root fracture occur more frequently [22]. Occlusal forces and predominantly functional and occasionally parafunctional forces tend to make cemented posts unstable in teeth that need coronal reconstruction [9].

Coronal preparation of the cast metal core after cementation may contribute to reducing its retention. Studies have proved the capacity of ultrasound vibration in facilitating removal of cemented posts in endodontically treated teeth [21, 23]. High-speed rotary instruments cause similar vibrations, which suggests that the layer of cement formed between the tooth and post may be fractured, thus compromising core retention [24].

The aim of this study was to investigate the effect of post-cementation waiting time for coronal preparation of core and the different types of cement had on cast metal core retention in root canals.

## 2. Methods and Materials

In this study, 60 extracted human canines of approximately the same size were collected and stored in aqueous 0.1% thymol. Each tooth was sectioned perpendicularly to its long axis 16 mm from the root apex, using a double-faced diamond disk number 7020 (KG Sorensen Ind. e Com. Ltda, Barueri, SP, Brazil) coupled to a straight handpiece at low speed, leaving a flat surface. The largest vestibular-lingual (VL) and mesiodistal (MD) diameters of the flat surfaces of the roots were recorded with a digital pachymeter (Mitutoyo Sul América LTDA., Suzano, SP, Brazil). These measurements were statistically compared to certify that the difference between the areas was not significant, and the discrepant teeth were eliminated.

The root canals were debrided conventionally with the K-type files up to number 45 (Maillefer Instruments, Ballaigues, Switzerland), irrigated with 5 mL of 2.5% sodium hypochlorite over 5 minutes, and filled using the lateral condensation technique with gutta-percha and a calcium hydroxide-based and eugenol-free endodontic cement (Sealer 26, Dentsply Materiais Odontológicos, Rio de Janeiro, RJ, Brazil). The teeth were sealed with temporary cement (Coltosol, Vigodent SA Ind. e Com., Rio de Janeiro, RJ, Brazil) and stored in distilled water in an oven at 37°C for 7 days.

To prepare the cores, 10 mm of root filling was removed with a Peeso bur (number 4 Maillefer Instruments, Ballaigues, Switzerland) at low speed controlled with a cursor. The root canals were cylindrical with 10 mm length and 1,3 mm diameter; they had the Peeso bur number 4 dimensions. To complete the preparation, a small channel was made on the internal vestibular wall of the root canal with a spherical carbide bur number 4 (KG Sorensen Ind. e Com. Ltda, Barueri, SP, Brazil) to guide the insertion of the cast core and prevent it from rotating.

The root canals and coronal surfaces were isolated with lubricant gel (KY, Johnson & Johnson Industrial Ltda, São José dos Campos, SP, Brazil) to mold the cores. Patterns were made of acrylic resin (Duralay, Reliance Dental Mfg. Co., USA) with the aid of prefabricated plastic posts (PinJet, Angelus, Londrina, PR, Brazil) for use in the 60 prepared teeth. In order to standardize the coronal portion of the cores, hollow crowns were used (Provjet-Angelus-Londrina, Paraná, Brazil). All the cores were made 1 mm short from the external margin of the flat coronal face of the tooth. The cores were cast in Ni-Cr alloy (Talladium do Brasil, Curitiba, PR, Brazil) and tested in the respective root canals to verify adaptation and were then subjected to airborne abrasion with 50 *μ*m aluminum oxide particles. A diamond tip was used to make scratches on the external root surface, perpendicular to the long axis, to provide additional retention of the root during tensile tenting.

Each tooth was fixed on the rod of a delineator (Bio Art, Art Equipamentos Ltda, São Carlos, SP, Brazil) with the aid of a number 4 Peeso reamer (Maillefer Instruments, Ballaigues, Switzerland) fitted to the canal so that the roots were perpendicular to the ground. They were then embedded in resin blocks. Proper devices were developed for the embedded specimens so that they would fit into the adaptor of the universal testing machine.

**Figure 1:**
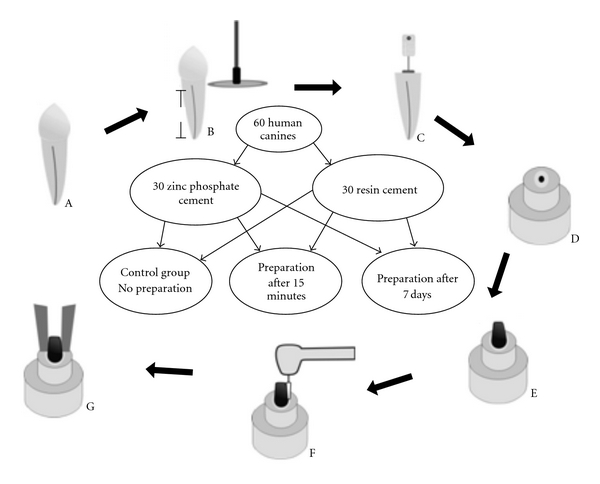
(A) Recently extracted canine. (B) Section of tooth. (C) Endodontic treatment. (D) Embedded sample. (E) Cemented cast metal core. (F) Coronal preparation of core. (G) Tensile test.

The teeth were cleaned with detergent (Tergensol, Inodon Laboratório, Porto Alegre, RS, Brazil) and the cast metal cores were also washed with detergent (Limpol, Bombril S/A, Abreu e Lima, PE, Brazil). The teeth were randomly divided into 2 cement groups (*n* = 30). The cast metal cores in the first group were cemented with zinc phosphate cement (SS WHITE Artigos Dentários Ltda, Rio de Janeiro, RJ, Brazil) using the technique described in Table 1, and in the second group they were cemented with resin cement (Panavia F, Kuraray Co., Osaka, Japan). The latter group was used in accordance with the manufacturer's recommendations. Table 1 describes the characteristics of the cements used and the manufacturers' instructions. The resin cement was placed only on the post. The zinc phosphate cement was placed on the post and also inserted into the canal with the aid of a Lentulo spiral (Maillefer Instruments, Ballaigues, Switzerland). After cementation, all the specimens were placed in a manual press and submitted to a 5 kgf pressure for 10 minutes to assure complete adaptation of the cast metal cores in the respective roots. The excess zinc phosphate cement was removed immediately after setting (10 minutes). In order to remove the excess resin cement, it was light activated for 10 seconds on the incisal margin of the core in the direction of the root. Each face of the tooth was then light activated for another 20 seconds with the light facing the core-tooth interface. To light activate the resin cement, a halogen light-curing appliance (Optilight LD MAX, Gnatus, Ribeirão Preto, SP, Brazil) with an irradiance of 450 mW/cm² was used. Each group of 30 teeth was subdivided into the following three groups (*n* = 10): Group 1 (control), time delay after setting; Group 2, coronal portion of post space prepared 15 minutes after cementation; and Group 3, coronal portion of post space prepared 7 days after cementation. The teeth were stored in a 100% humidity environment heated to 37°C during the storage time.  Figure 1 shows a diagram illustration presenting the experimental design of the tensile strength test.

The test specimens were fixed to a lathe and the metal cores were prepared by the same operator, using a tapered round trunk diamond tip (number 2135, KG Sorensen Ind. e Com. Ltda, Barueri, SP, Brazil) at high speed under cooling. The diamond tip was changed after every three preparations.

The coronal portion of the cores was prepared for 4 minutes : 3 minutes of axial preparation and 1 minute of incisal preparation. For the axial preparation, the diamond tip was placed so that it touched only the core in the direction of the long axis of the tooth, simulating preparation for a metal-ceramic crown. The diamond tip remained in contact with the core and the operator sought to exert constant pressure, going over all the faces of the core. For the incisal preparation, the diamond tip was placed forming an angle of 45° with the base.

All the test specimens were submitted to the tensile test 7 days after cementation. Tensile strength tests were performed in a testing machine (EMIC, são José dos Pinhais, PR, Brazil) at speed of 0.5 mm/min with a load cell of 2000 Kgf. The samples were placed and fastened to an adaptor at the base of the machine. A device on the active part of the machine was placed on the coronal portion of the cast metal core so that tensile force would be applied in the direction of the long axis of the tooth. As soon as the core was displaced, the test was interrupted and the displacement force value was recorded in the program. The tensile strength values were obtained in Kgf.

## 3. Results

Table 2 shows the results of the tensile strength test (mean and standard deviation) and the results of the Tukey test. The tensile strength values of the samples cemented with zinc phosphate cement were statistically higher than those of the samples cemented with Panavia F resin cement. None of the samples in either group were influenced by the post-cementation time interval waiting for coronal preparation of the core.

## 4. Discussion

Intraradicular posts are widely used to restore endodontically treated teeth that have insufficient coronal tooth structure to retain a definitive restoration [25, 26]. The use of prefabricated posts has become commonplace due to the satisfactory results, the reduction in clinical attendance time, and its quick application, which allows enhanced preservation of the tooth structure. However, a prefabricated retainer must adequately adapt to the prepared root canal; otherwise a cast post and core may be the treatment option [3]. 

Traditionally, zinc phosphate cement has been used to cement intraradicular retainers, although they have the disadvantage of lacking a bond both to the retainer and to the tooth structure [27]. In this regard, the retention provided by zinc phosphate is based mainly on mechanical interlocking. In addition to showing good results in retention tests, zinc phosphate cement has shown satisfactory performance in flexural tests and resistance to rotational forces [3, 6, 8, 10, 24]. On the other hand, resin cements have also been indicated for cementation of intraradicular retainers [6]. This type of cement could favor the retention of cast metal cores and prefabricated posts. Using resin cements was suggested mainly to strengthen the remainder of the tooth, due to the advancement of adhesive systems with properties of bonding to metals [18, 19, 23]. 

When comparing the means obtained by the resin luting agent and zinc phosphate cement in this experiment, it was observed that the latter showed better performance, corroborating other studies that also showed better results for zinc phosphate in comparison with resin composite used as luting agent in cast metal posts [9, 17, 20]. The hardening of zinc phosphate cement does not involve any reaction with the surrounding mineralized tissue or other restorative materials. Therefore, the main bond occurs by mechanical interlocking at the interfaces and not by chemical interactions. Due to this method of bonding, it may be inferred that the original adaptation of retainers before cementation and their irregularities may increase the retention of metal cores cemented with zinc phosphate cement.

Perfect adaptation of cast metal cores to the prepared canal results from the formation of a very thin film of cement. Zinc phosphate cement forms a thinner film than the resin cement, which may have contributed to the former's better performance observed in the present study. Due to frictional mechanical retention of the parts involved [6, 9], fracture of this thin film is made more difficult when there is a demand by traction.

The technique used to cement prosthetic parts may influence their final retention. The manufacturer of Panavia F does not recommend inserting the cement into the root canal with the aid of a Lentulo spiral because the cement setting may be altered due to the movement of particles, accelerating its setting time. This occurs due to the mixture of cement with the catalyzer (Ed Primer). However, the use of a Lentulo spiral to take the cement into the root canal when cementing cast posts, in addition to the cement applied on the post, prevents the presence of empty spaces inside the canal [8]. In this study, this technique was used for zinc phosphate cement, but not resin cement, which may have contributed to the more favorable result of the former than that obtained with the latter.

On the other hand, sodium hypochlorite, traditionally used as a chemical irrigant in endodontic treatment, may remove organic components from dentin, especially collagen, influencing the resin cement bonds to dentin. This might increase penetration of monomers into the demineralized dentinal structure; however, sodium hypochlorite dissociates into sodium chloride and oxygen. The oxygen present at the tooth-resin cement interface could inhibit cement polymerization and interfere in resin penetration into the dentinal tubules [14, 15]. In this regard, the use of sodium hypochlorite as an irrigant of canals may also have lowered the mean bond strength of the resin cement.

Another factor relative to endodontic treatment that might interfere with polymerization and resin cement bonding is the use of filling cements containing eugenol in their composition [16]. In order to avoid this interference, calcium hydroxide-based and eugenol-free filling cement was used.

The importance of adhesive systems in contemporary dentistry is unquestionable [1, 3, 6, 9]. The introduction of resin cements in dentistry has provided a significant improvement in the success rates of restorations retained by intraradicular retainers [12]. Thus, the good performance of resin cements in comparison with zinc phosphate cement has also been reported in the cementation of cast metal cores, prefabricated posts, indirect restorations, and fixed dentures [3, 5, 6, 21, 25, 28]. There are studies that have compared the retention of cast titanium cores cemented with zinc phosphate cement and resin cement and found no difference between the two types of cements [18, 19]. It should be pointed out that resin cements have some disadvantages; such as it is critical to use the correct technique, it is difficult to remove excess material when cementing, and it has a high cost [6].

Some studies have suggested that the lower values shown by resin cements in comparison with zinc phosphate cement are due to stress generated during polymerization shrinkage of resin cement, which causes its displacement from the dentinal surface as well as incomplete bonding. The high cavity factor (i.e., C factor) of a root canal and the impossibility of light from the light-curing unit penetrating deep enough into the canal may interfere and lead to the low tensile strength values [1, 15]. The use of a primer containing coinitiators, such as Panavia F ED Primer, is essential for the polymerization of resin cement when it is not exposed to light activation. Despite the capacity of the acid resin monomer present in Panavia F ED Primer to produce bonding between the resin composites and metal, the bond strength shown by Panavia F was not higher than the one shown by the zinc phosphate cement.

Some studies indicate that immature cement that is disturbed during setting time due to the preparation of the core may cause loss of cementation and, consequently, displacement of the post and it is suggested that the use of high-speed rotary instruments during the core repreparation is capable of causing vibration [26], damaging the cement film between the post and the root canal surface. Therefore, it is recommended that this type of preparation should not be performed immediately after cementation of intraradicular retainers [24, 29]. However, this study showed that the preparation of metal cores had no influence on the retention values obtained by tensile test. The differences in the results of these studies can be attributed to the variations in the study model that affect their clinical performance [24], particularly with regard to resin cements, technique sensitivity, and the difficulties with manipulation.

One of the relevant physical properties of the cement for retaining fixed dentures is its mechanical properties. Although zinc phosphate cement shows relatively low solubility in water, it is very resistant and capable of supporting elastic deformations even when it is used as a luting agent for restorations subject to high masticatory stresses [27].

In this study, the retention values of the zinc phosphate cement were much higher than those for the resin cement. There are some factors that may justify the results found, such as the methods of inserting the cement into the root canals, difficulty of polymerizing resin cement, cement film thickness, and the use of sodium hypochlorite to irrigate the root canals. Zinc phosphate cement is less sensitive to technique and is more retentive in the cementation of cast metal cores than resin cement and, therefore, it is recommended over resin cement. Coronal preparation of the core performed immediately after cementation did not diminish its retention, which favors the use of this clinical procedure due to its reduction in working time.

## 5. Conclusion

It may be concluded that cast metal cores cemented with zinc phosphate cement showed higher tensile strength values in comparison with resin cement. The preparation time of metal cores did not influence retention values obtained by the tensile test.

## Figures and Tables

**Table 1:** Characteristics of the cements used and the manufacturers' instructions.

Cement batch number manufacturer	Composition	Manufacturer's instructions
Zinc Cement Powder-0711008 Liquid-0010308 SS WHITE Artigos Dentários Ltda-RJ	Powder-zinc oxide, magnesium oxide, coloring agents Liquid-phosphoric acid, aluminum hydroxide, zinc oxide, distilled water	Recommended proportion: a small scoop measure of powder to 4 drops of liquid. Place powder on glass plate and divide it into multiple small parts. Shake the flask of liquid and drip liquid onto the plate. Begin mixing the powder and liquid immediately for 10 seconds per part, using a wide area of the plate. The powder was included incrementally to avoid excess heat development. Adequate consistency for cementation will be obtained when the mixture is creamy and leaves the spatula forming a sticky drop over a period of 60 to 90 seconds.
Panavia F 51203 Kuraray Co., Osaka, Japan	Paste A—10 methacryloyloxydecyl-hydrogen phosphate, hydrophobic and hydrophilic dimethacrylate, benzoyl peroxide, camphorquinone, colloidal silica Paste B—sodium fluoride, hydrophobic and hydrophilic dimethacrylate, diethanol-*p*-toluidine, Isopropyl T-sulphonate sodium benzoate, barium glass, titanium dioxide, colloidal silica	Tooth Preparation: mix 1 drop each of liquid A and B of ED Primer. Using a microbrush, apply the mixture in the root canal and wait 60 seconds. Remove excess primer with paper cone and conclude drying with light air jet. Paste Preparation: dispense equal quantities of pastes A and B on the manipulation block. The minimum quantity of paste should correspond to a half turn of the syringe. Mix the pastes for 20 seconds. Apply the paste to the post and on the coronal remainder. Insert the post into the canal and polymerize for 20 seconds.

**Table 2:** Tensile strength means in Kgf (standard deviations) between the groups and study times.

Times	Cement	
	Zinc phosphate	Resin cement
Control	31.65 (±12.10) Aa	8.65 (±3.08) Ba
15 minutes	33.26 (±10.44) Aa	8.48 (±4.71) Ba
7 days	27.21 (±6.50) Aa	7.48 (±4.33) Ba

Means followed by different letters (capitals letters in the lines and lower case letters in the columns) differ among themselves by the Tukey test (*P* < 0.05).
